# Boric Ester-Type Molten Salt via Dehydrocoupling Reaction

**DOI:** 10.3390/ijms151121080

**Published:** 2014-11-14

**Authors:** Noriyoshi Matsumi, Yoshiyuki Toyota, Prerna Joshi, Puhup Puneet, Raman Vedarajan, Toshihiro Takekawa

**Affiliations:** 1 School of Materials Science, Japan Advanced Institute of Science and Technology (JAIST), 1-1 Asahidai, Nomi, Ishikawa 923-1292, Japan; E-Mails: brothertobrother@gmail.com (Y.T.); joshi.prerna2011@gmail.com (P.J.); rupakagain2008@gmail.com (P.P.); raman@jaist.ac.jp (R.V.); 2 Advanced Materials Laboratory, Nissan Motor Co., Ltd., 1 Natsushima-cho, Yokosuka-shi, Kanagawa 237-8523, Japan; E-Mail: t-takekawa@mail.nissan.co.jp

**Keywords:** molten salts, lithium ion, organoboron compounds, lithium transference number

## Abstract

Novel boric ester-type molten salt was prepared using 1-(2-hydroxyethyl)-3-methylimidazolium chloride as a key starting material. After an ion exchange reaction of 1-(2-hydroxyethyl)-3-methylimidazolium chloride with lithium (*bis*-(trifluoromethanesulfonyl) imide) (LiNTf_2_), the resulting 1-(2-hydroxyethyl)-3-methylimidazolium NTf_2_ was reacted with 9-borabicyclo[3.3.1]nonane (9-BBN) to give the desired boric ester-type molten salt in a moderate yield. The structure of the boric ester-type molten salt was supported by ^1^H-, ^13^C-, ^11^B- and ^19^F-NMR spectra. In the presence of two different kinds of lithium salts, the matrices showed an ionic conductivity in the range of 1.1 × 10^−4^–1.6 × 10^−5^ S cm^−1^ at 51 °C. This was higher than other organoboron molten salts ever reported.

## 1. Introduction

In recent years, lithium ion batteries (LIB) [1,2] have taken a very important role as energy storage devices with the development and spread of high performance microelectronics and zero-emissions vehicles. However, lithium-ion batteries are not bereft of demerits. For instance, the highly flammable electrolyte has always been a subject of concern and has been a topic of continuous research. Meanwhile, ionic liquids (ILs) [3,4,5] have also been attracting immense interest as electrolytes for a variety of ionic devices, because of their high ionic conductivity, designability and non-flammable property. A few disadvantages associated with ILs have kept them away from manufacturing scale development and has kept research afresh in this topic. One such disadvantage is the migration of both anion and cation under the potential gradient, and the selective transport of target cation is not straight forward. In the search for a solution to these problem, previously, the synthesis of a few novel organoboron molten salts [6] bearing tertiary boron was proven to be an efficient electrolyte. A maximum lithium ion transference number reached as high as 0.7 for those systems. It was shown that the highly Lewis acidic alkylborane group trapped anions more efficiently than boric ester groups did. Moreover, polymer homologues of organoboron molten salts prepared by hydroboration polymerization showed *t*_Li+_ of 0.87 at 30 °C [7]. Furthermore, it was found that anion trapping in ionic liquid-based media worked more efficiently than was observed in polyether-based media. However, the ionic conductivity of such organoboron molten salts was low, ranging in the order of 10^−5^–10^−6^ S cm^−1^ at 50 °C.

As an alternative approach to improve the lithium transference number of electrolytes, the design of zwitterionic molten salts has been examined by H. Ohno *et al*. [8,9]. Organoboron zwitterionic molten salt bearing a highly dissociable pentafluorophenylborate structure [10] showed a lithium transference number of 0.69 with ionic conductivity of 3.0 × 10^−5^ S cm^−1^, which was comparable to alkylborane-type molten salt. So far, boron incorporated into lithium ion conductive electrolytes was found to be effective for any type of electrolyte, such as solid polymer electrolytes, ionic liquids (molten salts) and polymer ion–gel electrolytes.

Boron incorporation in electrolytes [11,12,13,14,15,16,17,18,19,20,21] generally leads to either: (1) promotion of lithium salt dissociation; or (2) an enhanced lithium transference number via anion trapping by the boron atom. Usually, in the case of polymer/salt hybrids [22,23] in which the anion structure was immobilized onto the polymer framework, enhancement of the lithium transference number always results in a decrease of ionic conductivity. However, an important point of organoboron electrolytes, in principle, is that both the lithium transference number and ionic conductivity can be enhanced at the same time, if the strength of the Lewis acid–anion interaction is adequately tuned.

**Scheme 1:**
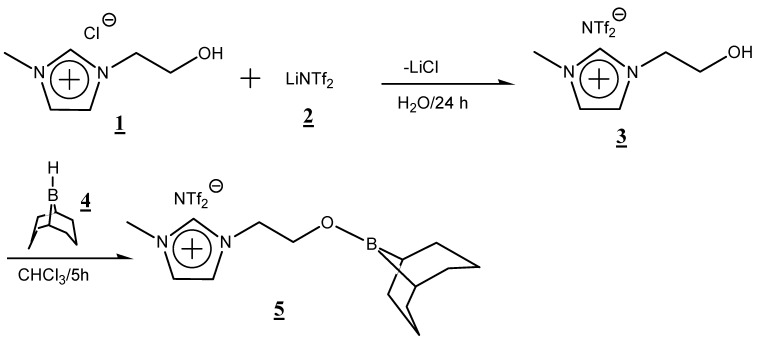
Synthesis of novel organoboron molten salt derived from 1-(2-hydroxyethyl)-3-methylimidazolium chloride.

In the search for such an attractive system, in the present work, a novel organoboron molten salt **5** was synthesized using 9-borabicyclo[3.3.1]nonane (9-BBN) and 1-(2-hydroxyethyl)-3-methyl imidazolium chloride as starting materials (Scheme 1). The synthesized organoboron molten salt was well characterized using ^1^H-, ^13^C-, ^19^F- and ^11^B-NMR. The intrinsic characteristics, such as the conductivity and thermal stability of the obtained organoboron molten salt, were evaluated.

## 2. Results and Discussion

After the ion exchange reaction of 1-(2-hydroxyethyl)-3-methylimidazolium chloride (**1**) with lithium (*bis*-(trifluoromethanesulfonyl) imide) (LiNTf_2_) (**2**), the resulting 1-(2-hydroxyethyl)-3-methylimidazolium NTf_2_ (**3**) was reacted with an excess amount of 9-BBN (**4**) to give the desired organoboron molten salt in an 85% yield. The molten salt, obtained as a white soft solid, was soluble in common organic solvents, such as chloroform, methanol, dimethyl sulfoxide (DMSO) and so forth, and the structure was supported by ^1^H-, ^13^C-, ^11^B- and ^19^F-NMR spectra.

**Figure 1:**
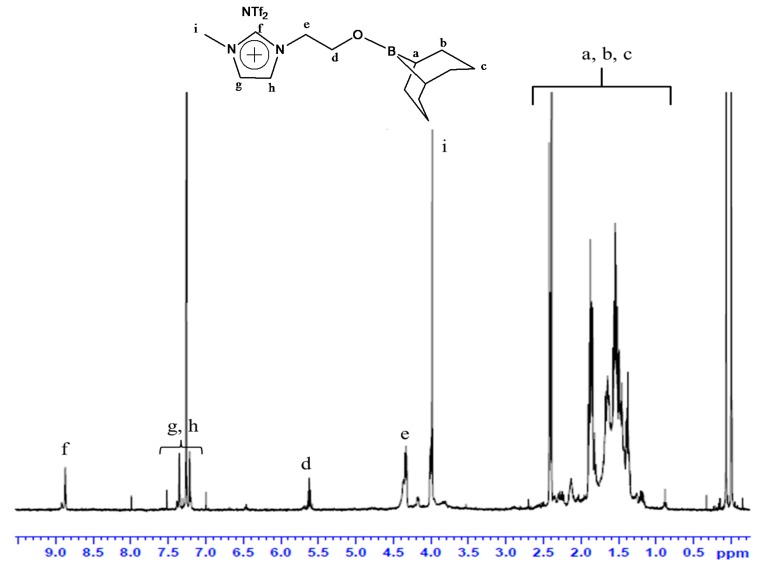
^1^H-NMR of **5** (solvent: CDCl_3_; 10%; r.t.; TMS; 400 MHz).

**Figure 2:**
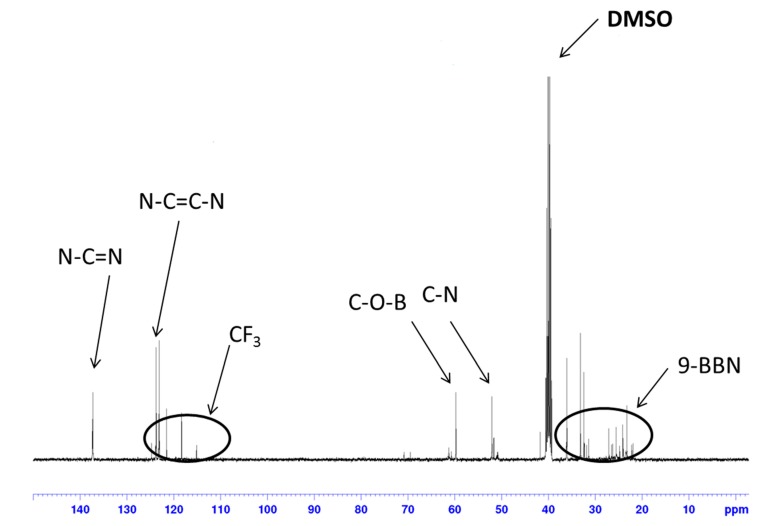
^13^C-NMR of **5** (solvent: DMSO; r.t.; 100 MHz). 9-BBN, 9-borabicyclo[3.3.1]nonane.

In the ^1^H-NMR spectrum of organoboron molten salt measured in CDCl_3_ (Figure 1), the peaks due to the 9-BBN structure were observed in 1.26–2.50 ppm. Moreover, the chemical shift of the methylene proton adjacent to the hydroxyl group (4.33 ppm, NCH_2_CH_2_OH) was shifted after dehydrocoupling reaction (5.62 ppm, NCH_2_CH_2_OBR_2_). The integration ratios for all of the peaks were in good agreement with that of the expected structure. The ^13^C-NMR spectrum (Figure 2) was also in accord with the expected structure.

**Figure 3:**
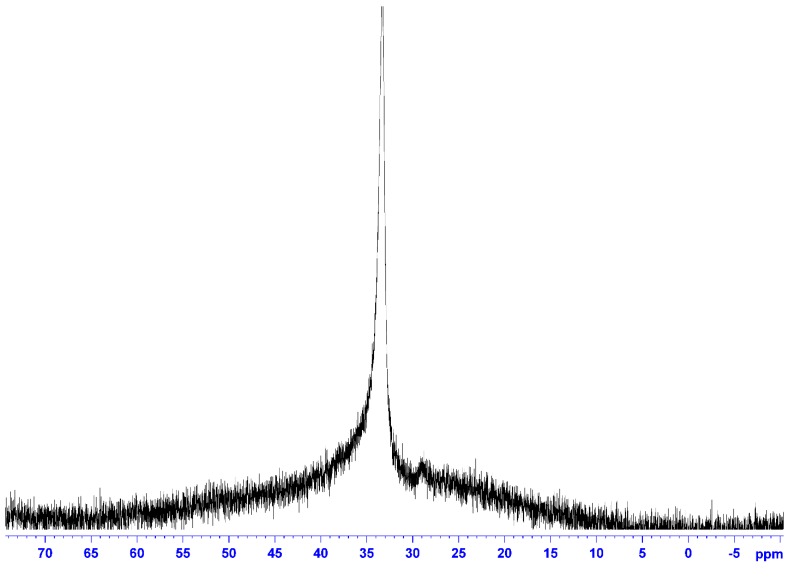
^11^B-NMR of **5** (solvent: CDCl_3_; r.t.; external standard: B(OCH_3_); 128 MHz).

**Figure 4:**
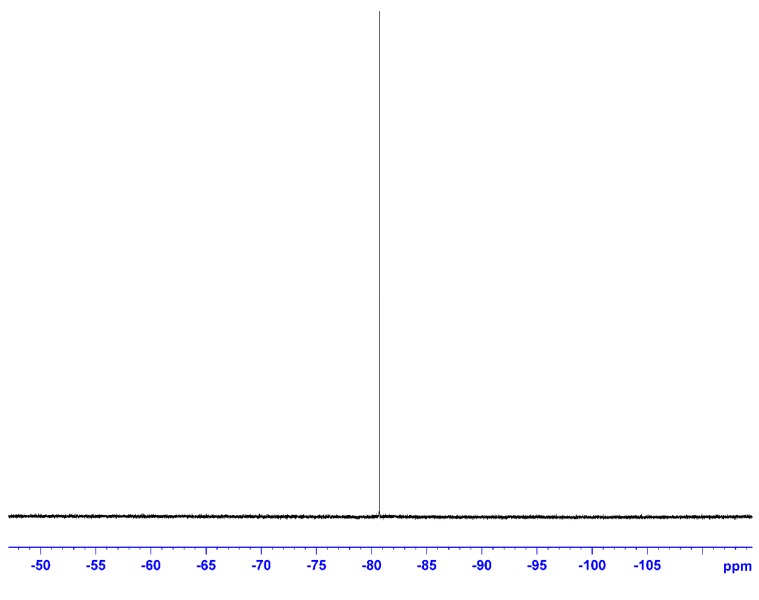
^19^F-NMR of **5** (solvent: CDCl_3_; r.t.; external standard: C_6_H_5_CF_3_; 376 MHz).

The ^11^B-NMR spectrum (Figure 3) was also measured in CDCl_3_ using B(OCH_3_)_3_ as the external standard. The only peak due to O–BR_2_ was observed at 33.3 ppm, showing that the boron atom had a single chemical environment. The ^19^F-NMR spectrum (Figure 4) measured using C_6_H_5_CF_3_ as the external standard also showed a single main peak at −80.7 ppm. This also supported the incorporation of the NTf_2_ anion in the ionic liquid structure.

**Figure 5:**
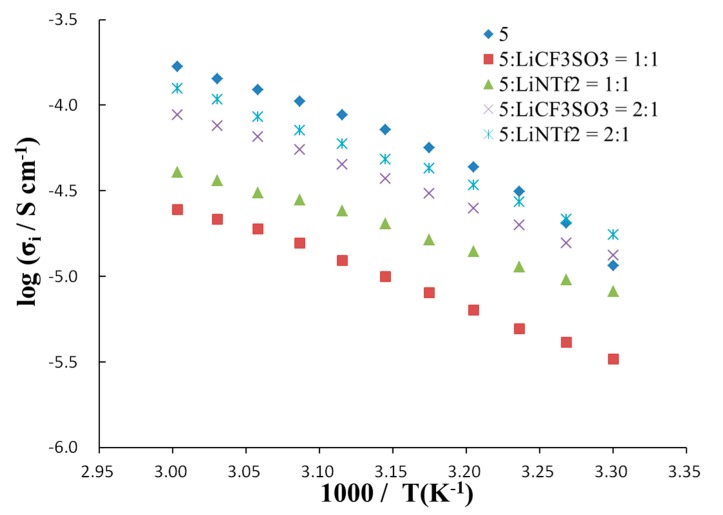
Temperature dependence of ionic conductivity for novel organoboron molten salt in the presence or absence of lithium salt.

The effect of temperature on ionic conductivity (Figure 5) was studied by AC-impedance measurements after a designated amount of lithium salt (lithium trifluoromethanesulfonate (LiCF_3_SO_3_)_3_ or LiNTf_2_) was added to the molten salts. The ionic conductivity observed was in the range of 1.1 × 10^−4^–1.6 × 10^−5^ S cm^−1^ at 51 °C. All of the systems showed lower ionic conductivity compared with bulk molten salt **5**, which is free of lithium salt. This should be due to the increase in viscosity with the addition of lithium salt. The ion conductive matrices containing LiNTf_2_ showed higher ionic conductivity in comparison with that containing LiCF_3_SO_3_. It was observed that the samples containing a **5**:Li ratio of 2:1 (molar ratio) exhibited higher ionic conductivity in comparison with the ratio of **5**:Li = 1:1 (molar ratio).

**Figure 6:**
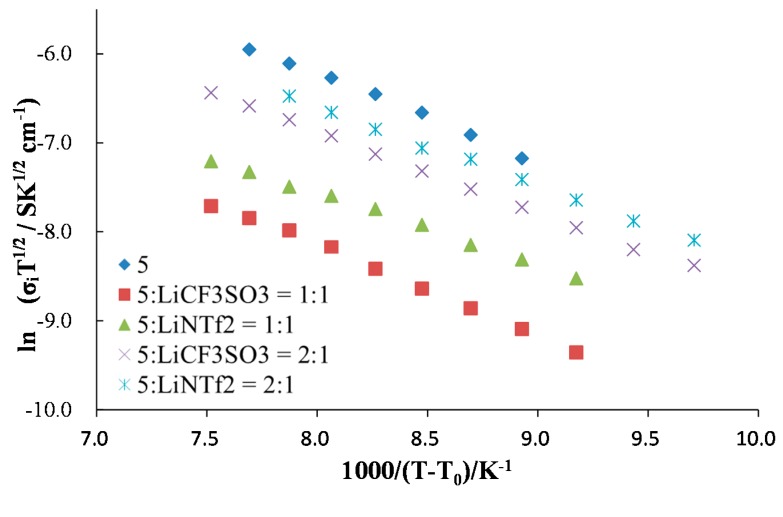
VFT (Vogel–Fulcher–Tammann) plots for ion transport matrices.

In order to obtain further information on the ion conductive behavior, the temperature dependence of ionic conductivity was fitted with the VFT (Vogel–Fulcher–Tammann) equation (Equation (1), Figure 6) [24,25,26]. The VFT parameters calculated are listed in Table 1. The VFT parameter B, corresponding to the activation energy of ion transport for matrices, was lower in the presence of LiNTf_2_. This indicates that the plasticizing effect of NTf_2_ anion was responsible for higher ionic conductivity for these systems. Under higher lithium ion concentration, the parameter B for LiCF_3_SO_3_-based systems increased. This should be due to the higher viscosity under the higher LiCF_3_SO_3_ concentration. On the contrary, the LiNTf_2_-based system did not show an increase in the parameter B under a higher lithium salt concentration, possibly because of the plasticizing effect of the NTf_2_ anion. However, both systems showed a decrease in ionic conductivity with increasing lithium salt concentrations, due to the decreased number of carrier ions (decreased dissociation degree of lithium salt) under the significantly high ionic strength of the matrices. The values of B were in accordance with the viscosity of the Compound **5** as a soft solid.


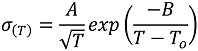
(1)

From the TGA measurements, the organoboron molten salt was found to be stable, even at temperatures higher than 100 °C. *T*_50%_ was observed to be around 430 °C for all the samples.

## 3. Experimental Section

### 3.1. Instruments and Materials

1-(2-Hydroxyethyl)-3-methylimidazolium chloride was purchased from Kanto Chemical Co. Ltd. (Tokyo, Japan) and used without further purification. Lithium (*bis*-(trifluoromethanesulfonyl) imide) (LiNTf_2_) was also purchased from Kanto Chemical Co. Ltd. and used as received. Lithium trifluoromethanesulfonate (LiCF_3_SO_3_) was purchased from Wako Co. Ltd. (Osaka, Japan) and used as received. Lithium sheets were purchased from Honjo Chemical Co. Ltd. (Osaka, Japan). The 0.5 M THF solution of 9-borabicyclo[3.3.1]nonane was purchased from Across Co. Ltd. (Geel, Belgium). Dehydrated organic solvents (diethyl ether, chloroform, *n*-hexane, tetrahydrofuran) were purchased either from Kanto Chemical Co. Ltd. or Wako Co. Ltd. and used as received. All of the reactions were carried out under a nitrogen atmosphere.

^1^H- and ^11^B-NMR spectra were recorded on a Bruker Avance III 400 (Bruker, Billerica, MA, USA). After adding the appropriate amount (molar ratio) of LiNTf_2_ or lithium trifluoromethanesulfonate (LiCF_3_SO_3_, Kanto Chemical Co. Ltd.), the ionic conductivity of the ion conductive matrices was measured with a complex-impedance gain-phase analyzer (Solartron model 1260; Schlumberger, Houston, TX, USA) under the frequency range from 1 Hz to 1 MHz. IR spectra were measured on a JASCO FT/IR-4100 spectrometer (Tokyo, Japan). Thermogravimetric analysis was made on a PerkinElmer TGA7 (PerkinElmer, Waltham, MA, USA).

**Table 1:** VFT parameters of ion transport matrices.

Sample	*A* (S cm^−1^ K^1/2^)	*B* (K)	*T*_0_ (K)	*R* ^2^
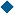	5.27	985	200	0.996
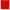	0.954	1,013	200	0.998
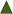	0.302	796	200	0.997
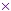	1.47	907	200	0.999
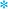	1.54	879	200	0.999

### 3.2. Synthesis of Organoboron Molten Salt

#### 3.2.1. Ion Exchange Reaction of 1-(2-Hydroxyethyl)-3-methylimidazolium Chloride

To an aqueous solution of 1-(2-hydroxyethyl)-3-methylimidazolium chloride (2.51 g, 15.3 mmol), 5.32 g (18.4 mmol) of LiNTf_2_ was added, and the resulting mixture was stirred at room temperature for 24 h. An Oily liquid was spontaneously separated from the aqueous layer. After removing the aqueous layer by decantation, the oily product was purified by reprecipitation into diethyl ether. After removing the solvent under a reduced pressure, the desired compound, 1-(2-hydroxyethyl)-3-methylimidazolium NTf_2_, was obtained as a colorless soft solid (5.29 g, 13.0 mmol, 84% yield).

^1^H-NMR (CDCl_3_, δ, ppm): 3.82 (1H, CH_2_CH_2_OH), 3.99 (3H, CH_3_), 4.19 (2H, –CH_2_CH_2_OH), 4.33 (2H, –CH_2_CH_2_OH), 7.21, 7.35 (2H, –NCHCHN–), 8.88 (1H, –NCHN–).

#### 3.2.2. Dehydrocoupling Reaction of 1-(2-Hydroxyethyl)-3-methylimidazolium NTf2

To a chloroform (15 mL) solution of 1-(2-hydroxyethyl)-3-methylimidazolium NTf_2_ (4.10 g, 10.1 mmol), 38 mL of 0.5 M THF solution of 9-borabicyclo[3.3.1]nonane (9-BBN) (19.1 mmol) were added at 0 °C, and the resulting mixture was stirred for 5 h. After removing the solvent under a reduced pressure, excess 9-BBN was removed by washing with *n*-hexane several times. The product was thoroughly dried under a reduced pressure before use. The desired compound was obtained as a soft white solid (4.52 g, 8.57 mmol, 85% yield). ^1^H-NMR (CDCl_3_, δ, ppm) 1.26–2.50 (20H, 9-BBN), 3.98 (3H, CH_3_), 4.33 (2H, –NCH_2_CH_2_O), 5.62 (2H, –NCH_2_CH_2_OB–), 7.21, 7.35 (2H, –NCHCHN–), 8.87 (1H, –NCHN–). ^13^C-NMR (DMSO-d_6_, δ, ppm) 23.3–33.2 (9-BBN), 36.1, 52.1, 59.8, 119.4 (q, CF_3_), 123.1–123.8 (N–C=C–N), 137.3 (N–C=N). ^11^B-NMR (CDCl_3_, δδ, ppm) 33.3.

## 4. Conclusions

Novel organoboron molten salt using 1-(2-hydroxyethyl)-3-methylimidazolium chloride as a starting material was synthesized in an 85% yield. The structure was supported by ^1^H-, ^13^C-, ^11^B- and ^19^F-NMR spectra. A maximum ionic conductivity of 1.1 × 10^−4^ S cm^−1^ was observed for the sample with LiNTf_2_ salt. This system showed higher ionic conductivity than any other organoboron molten salt reported so far. All of the samples showed good thermal stability with *T*_50%_ around 430 °C. The high thermal stability and the high ionic conductivity render the new organoboron-IL with desirable characteristics as a lithium ion transport material.
